# Examining Work Engagement in Integrated Nursing-Care Service Wards: Insights from Structural Equation Modeling

**DOI:** 10.1155/2024/5670381

**Published:** 2024-08-16

**Authors:** Ok Yeon Cho, Seon-Heui Lee, Soyoung Yu

**Affiliations:** ^1^ Graduate School College of Nursing Gachon University, 191 Hambakmoero, Yeonsu-gu, Incheon 21936, Republic of Korea; ^2^ College of Nursing Research Institute of AI and Nursing Science Gachon University, 191 Hambakmoero, Yeonsu-gu, Incheon 21936, Republic of Korea; ^3^ College of Nursing CHA University, 120, Pocheon, Gyeonggi-do, Republic of Korea

## Abstract

**Background:**

In 2016, the South Korean Integrated Nursing-Care Service, covered by national insurance, was initiated, with a particular focus on cancer-oriented units. Integrated Nursing-Care Service Wards denote facilities wherein nursing professionals deliver holistic care, in the absence of paid informal caregivers (hereafter will be called caregiver).

**Aim:**

This study, framed within Demerouti's Job Demands-Job Resources Model, aimed to analyze variables influencing nurses' work engagement in Integrated Nursing-Care Service wards.

**Methods:**

From April to June 2022, 375 participants working at three certified tertiary hospitals operating Integrated Nursing-Care Service wards completed the survey. Of the 400 distributed questionnaires, 375 were used for analysis, resulting in a response rate of 93.75%. The remaining 25 questionnaires were excluded due to insufficient responses. Job demands, job resources, and personal resources were assigned as exogenous variables that predicted burnout and work engagement of nurses, while burnout and work engagement were assigned as endogenous variables. In this model, 32 hypotheses were established, and to verify the hypotheses, the direct effect of each exogenous variable on work engagement and the indirect effect through burnout as a medium were analyzed.

**Results:**

Burnout partially mediated the impact of exogenous variables on work engagement. The subfactors revealed partial mediation between emotional labor and work engagement, full mediation for satisfaction with the recognition from patients and caregivers, and partial mediation for resilience.

**Conclusion:**

Emotional labor had the highest impact on nurses' burnout in Integrated Nursing-Care Service wards, followed by resilience and satisfaction with the recognition from patients and caregivers. Nurses' burnout, work environment, emotional labor, work overload, and resilience significantly influenced their work engagement. *Implications for Nursing Management*. The results of this study are useful as basic data for research on intervention programs that reduce burnout and increase nurses' work engagement in Integrated Nursing-Care Service wards.

## 1. Introduction

Changes in family and social structures, such as population aging, the rise of chronic diseases, and nuclear families, are phenomena that occur in many countries worldwide [1]. In South Korea, the social activities and demands of family members have led to a trend of shifting direct care from family members to paid informal caregivers when patients are hospitalized. Caregivers help patients with their daily activities on behalf of their families or play a role in patient safety under the direction and supervision of nurses [2]. South Korea has a nursing workforce of 8.8 nurses per 1,000 people, which is 1.0 less than the Organization for Economic Co-operation and Development (OECD) average (9.8) [3]. The ratio of patients per nurse in the US is 5.3, while the ratio in South Korea is nearly 16.3 patients per nurse [4], which is still inadequate, and the burden of family caregiving and care costs continues to increase due to the lack of nursing staff and the hiring of private caregivers.

In 2013, the government expanded the nursing staff to include nurses and nursing assistants to provide nursing care to patients who were previously cared for by family members or private paid caregivers [5]. In 2016, the government renamed the program as the Nursing and Care Integration Service and gradually expanded the initiative to include not only small- and medium-sized hospitals but also general hospitals [6]. The Integrated Nursing-Care Service is a comprehensive nursing care service that provides hospitalized patients with adequate nursing care, even if their guardians are not present, by securing adequate nursing staff, introducing a team care system, and improving the ward environment. In principle, all nursing services provided to hospitalized patients are provided by nurses and nursing assistants in medical institutions, and the system aims to improve the quality of hospitalized medical services and reduce the caregiving burden on guardians. On the patient side, providing Integrated Nursing-Care Service has led to positive changes in quality, including increased satisfaction with the high quality of care, improved intentions to return for care due to better ward environment, and decreased incidence of falls, pressure ulcers, and infections, which are patient safety indicators [7].

On the other hand, in terms of nurses, who are the mainstay of the core workforce, there are also negative aspects, such as the burden of increased workload and emphasis on the importance of nurses' role, as they are required to provide 24-hour care for patients in close proximity without a guardian or caregiver [5]. In addition, there are many cases of job stress leading to burnout among nurses in Integrated Nursing-Care Service wards due to distrust from patients and caregivers who are accustomed to receiving care from family members and caregivers and demands beyond the scope of nursing services [8]. Excessive job stress due to changes in traditional nursing duties and increased responsibilities can lead to negative work attitudes and self-concept, leading to burnout [9].

Nurse burnout negatively affects job satisfaction in the long run and reduces the quality of healthcare services provided by nurses [10]. Therefore, interventions and strategies are needed to decrease burnout and increase work engagement among nurses in Integrated Nursing-Care Service wards. Burnout refers to a state of physical, emotional, and mental exhaustion caused by chronic stress during the workday for members of an organization with frequent contact with others [11]. It can lead to job dissatisfaction and increased errors in nursing care due to formal work performance, which in turn can lead to decreased patient and caregiver satisfaction and nurse turnover [12]. Strengthening nurses' resilience is an important factor that can help them overcome the difficulties in work performance caused by burnout, and improve the quality of care. Resilience, an internal coping resource, is the ability to respond flexibly and adapt to negative factors [12], and individuals with higher levels of resilience may be able to overcome the negative effects of workplace adversity and challenges and consequently experience less burnout [13].

In contrast to burnout, work engagement refers to a positive and fulfilling attitude toward work and motivating the nursing workforce [14]. Individuals with high work engagement have a strong sense of belonging to the organization, engage in their work to seek positive feedback from supervisors and the organization, and have a strong passion for activities outside of their role. Work engagement also affects nurses' personal resources, such as resilience and self-esteem [15].

Burnout and work engagement in nursing can also be explained by the Job Demands-Resources model (JD-R model), which explains burnout and work engagement from an organizational perspective rather than an individual perspective and divides job-related factors into job demands and job resources. Xanthopoulou et al. [16] extended the JD-R model by adding personal resources to it. Later, Keyko et al. [17] extended the model to develop the Nursing Job Demands-Resources (NJD-R) model applicable to professional nursing practices. According to their findings, work engagement exists in the organizational environment and at the work level, and the positive outcomes of work engagement can improve performance for the organization, increase value for the nurses, and decrease negative outcomes within the healthcare organization. The NJD-R model, derived from this research, suggests opportunities to promote nurses' work engagement, providing a foundational framework for understanding nursing practice, conducting direct nursing research, and guiding practices and policies [17].

Therefore, this study aimed to identify the effects of burnout and work engagement on nurses in Integrated Nursing-Care Service wards and explore ways to decrease burnout and increase work engagement in Integrated Nursing-Care Service wards by identifying factors that affect work engagement through the mediation of burnout and identifying pathways between factors. In this study, burnout was examined as an outcome influenced by various job demands and resources. This perspective is crucial as it allows us to understand how burnout impacts job satisfaction and the quality of healthcare services. By treating burnout as a result, we can better analyze its underlying causes and develop strategies to mitigate its effects on healthcare professionals and the care system as a whole.

## 2. Materials and Methods

### 2.1. Design and Sampling

**Figure 1:**
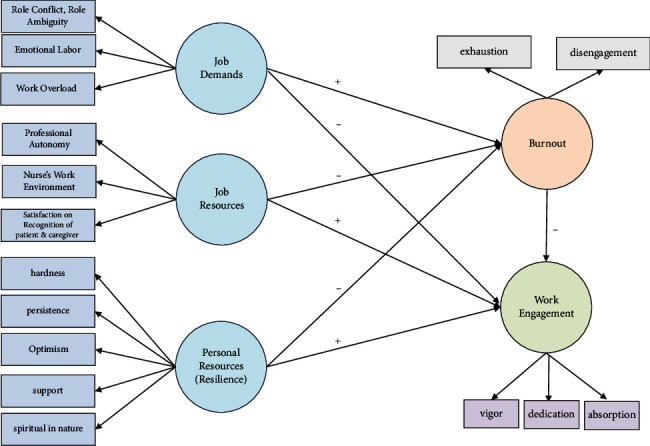
The conceptual map of study variables.

This is a descriptive research study based on the JD-R model (Figure 1). It consists of seven exogenous variables (role conflict and role ambiguity, emotional labor, work overload, professional autonomy, nurses' work environment, satisfaction with the recognition from patients and caregivers, and resilience) and two endogenous variables (burnout and work engagement), with burnout as a parameter. In this context, “burnout as a parameter” means that burnout is used as an intermediary variable that mediates the relationship between the exogenous variables and the endogenous variables. This mediating role allows us to examine how burnout influences the impact of these variables on work engagement. The paths between the variables were hypothesized to be role conflict and role ambiguity, emotional labor, work overload, professional autonomy, nurses' work environment, satisfaction with the recognition from patients and caregivers, resilience affecting burnout and work engagement, and burnout affecting work engagement.

### 2.2. Data Collection

Data were collected from April 27 to June 24, 2022, from nurses with at least 1 year of work experience in three tertiary general hospitals and who experienced similar levels of work intensity in Integrated Nursing-Care Service wards in Incheon and Bucheon, South Korea, and understood the purpose of the study and agreed to participate. After obtaining prior consent from the head of the nursing department of the hospital, the researcher sought cooperation from the head nurse of the ward, explained the purpose of the study, obtained written consent, and administered the questionnaire. To reduce sampling errors in structural equations, a sample size of 200–400 is required, regardless of the size of the model [18]. Accordingly, 400 questionnaires were distributed, and 375 questionnaires were analyzed after excluding those who responded dishonestly.

### 2.3. Measures

Participant characteristics included age, gender, education, position, clinical experience, experience in Integrated Nursing-Care Service wards, and length of service in Integrated Nursing-Care Service wards.

Job demands included role conflict and role ambiguity, emotional labor, and work overload; job resources were professional autonomy, nurses' work environment, satisfaction with the recognition from patients and caregivers; personal resources were resilience; and endogenous variables were burnout and work engagement.

#### 2.3.1. Job Demands

*(1) Role Conflict and Role Ambiguity*. The questionnaire developed by Rizzo and Lirtzman [19] and adapted by Kim [20] has a total of 10 questions rated on a 5-point Likert scale, with higher scores indicating higher role conflict and role ambiguity. In this study, the reliability of role conflict was Cronbach's *ɑ* 0.81, and the reliability of role ambiguity was Cronbach's *ɑ* 0.79.

*(2) Emotional Labor*. Emotional labor refers to the process of managing feelings and expressions to fulfill the emotional requirements of a job [21]. It includes factors such as the frequency of emotional expression, caution in emotional expression, and emotional dissonance, measured by a tool developed by Kim [22] based on the research of Morris and Feldman [21]. The tool comprises a total of nine subfactors, including three questions each on the frequency of emotional expression, caution in emotional expression, and emotional dissonance. Participants rated these subfactors on a 5-point Likert scale, with higher scores indicating elevated levels of emotional labor. The reliability of the tool in this study was assessed using Cronbach's alpha, yielding a value of 0.83.

*(3) Work Overload*. The work overload measure, a component of the Work-Job Demands subscale within the Questionnaire on the Experience and Evaluation of Work (QEEW) 2.0 tool, was developed by Van Veldhoven et al. [23] and translated by Lim [24]. This measure consists of six questions, and participants rate their responses on a 4-point Likert scale. Higher scores on this scale indicate a greater perception of work overload. The reliability of the tool in this study was assessed using Cronbach's alpha, yielding a value of 0.88.

#### 2.3.2. Job Resources

*(1) Professional Autonomy*. The Professional Autonomy Scale developed by Schutzenhofer [25], adapted by Han [26], and modified by Kim [27] has a total of six questions rated on a 5-point Likert scale, with higher scores indicating higher levels of professional autonomy. The reliability of this study was Cronbach's *ɑ* 0.76.

*(2) Nurses' Work Environment*. The Korean Work Environment Scales for Clinical Nurses (KWES-CN), developed by Kim et al. [28] and modified and supplemented by Shim [29], have a total of 18 questions rated on a 4-point Likert scale, with higher scores indicating a better nurses' work environment. The reliability of this study was Cronbach's *ɑ* 0.86.

*(3) Satisfaction with Recognition from Patients and Caregivers*. The Job Satisfaction Tool developed by Paula et al. [30], translated by Han et al. [26], and used by Park [31] and the Volunteer Satisfaction Tool of Choi [32] reorganized, modified, and supplemented by Shim [29] have a total of five questions rated on a 5-point Likert scale, with higher scores indicating higher satisfaction with the recognition from patients and caregivers. The reliability of this study was Cronbach's *ɑ* 0.87.

#### 2.3.3. Personal Resources

*(1) Resilience*. The Connor–Davidson Resilience Scale (CD-RISC), developed by Connor and Davidson [33] and adapted and validated by Baek [34] as the K-CD-RISC, has a total of 25 questions rated on a 5-point Likert scale, with higher scores indicating higher resilience. CD-RISC indeed provides subscales, measuring resilience through five distinct factors: hardiness, persistence, optimism, support, and spirituality [34]. Each subscale is assessed through specific items on the scale. Hardiness reflects the ability to endure difficult conditions. Persistence indicates the capacity to continue despite adversity. Optimism measures a general positive outlook on life and the future. Support evaluates the perceived availability of social support, and spirituality captures a sense of purpose and faith in something greater. The reliability of this study was Cronbach's *ɑ* 0.92.

#### 2.3.4. Burnout

The Oldenburg Burnout Inventory (OLBI), developed by Demerouti and Nachreiner [35] and translated by Choi [36], has a total of six questions rated on a 5-point Likert scale, with higher scores indicating higher levels of burnout. The reliability of this study was Cronbach's *ɑ* 0.76.

#### 2.3.5. Work Engagement

Work engagement is defined as a positive, fulfilling, work-related state of mind characterized by vigor, dedication, and absorption [37]. It is measured using the Dutch Utrecht Work Engagement Scale (UWES-9). The Dutch Utrecht Work Engagement Scale (UWES-9), developed by Schaufeli et al. [37] and translated by Baek [38], has a total of nine questions rated on a 7-point Likert scale, with higher scores indicating higher work engagement. The reliability of this study was Cronbach's *ɑ* 0.91.

### 2.4. Data Analysis

Descriptive statistics and Pearson's correlation coefficient analysis were conducted using SPSS 25.0, and the fitness of the hypothetical model, path coefficient, and effect were verified using AMOS 23.0.

To verify the suitability of the model, absolute fit indices (*χ*^2^, *χ*^2^/df), root mean square residual (RMR), goodness-of-fit index (GFI), adjusted goodness-of-fit index (AGFI), root mean square error of approximation (RMSEA), Tucker–Lewis index (TLI), and comparative fit index (CFI) were used for the analysis.

The significance of the path coefficient of the structural model was verified using the unstandardized coefficient (B), standardized coefficient (*β*), standard error (SE), and *p* value, and the explanatory power of the endogenous variables was confirmed using square multiple correlation (SMC).

### 2.5. Ethical Considerations

After obtaining approval from the Institutional Review Board of Gachon University (IRB approval number: 1044396-202201-HR-027-01), written consent was obtained from all the participants, and the study contents were explained to them. The written consent form included information on the purpose of the study, data collection methods, autonomy to participate in or withdraw from the study, confidentiality of information and interests, discomfort, and time required to complete the questionnaire. A self-administered questionnaire was administered to those who voluntarily agreed to participate in writing. All the nurses who participated in the study were offered a small gift as a token of appreciation.

## 3. Results

### 3.1. General Characteristics

The average age of the nurses was 29.90 years (±6.32), approximately 95.7% of the total sample was female, and four-fifths of the nurses had a bachelor's degree in nursing. More than 80% of the positions were general nurses, with an average clinical experience of 7.32 (±6.76) years, 107 (35.7%) had 1-2 years of experience in nursing and Integrated Nursing-Care Service wards, 150 (50.0%) had 3–5 years of experience, and 43 (14.3%) had more than 6 years of experience in nursing and Integrated Nursing-Care Service wards, and the average number of years of experience in nursing and integrated care units was 3.33 (±1.62) (Table 1).

### 3.2. Validation Results of the Research Model

**Figure 2:**
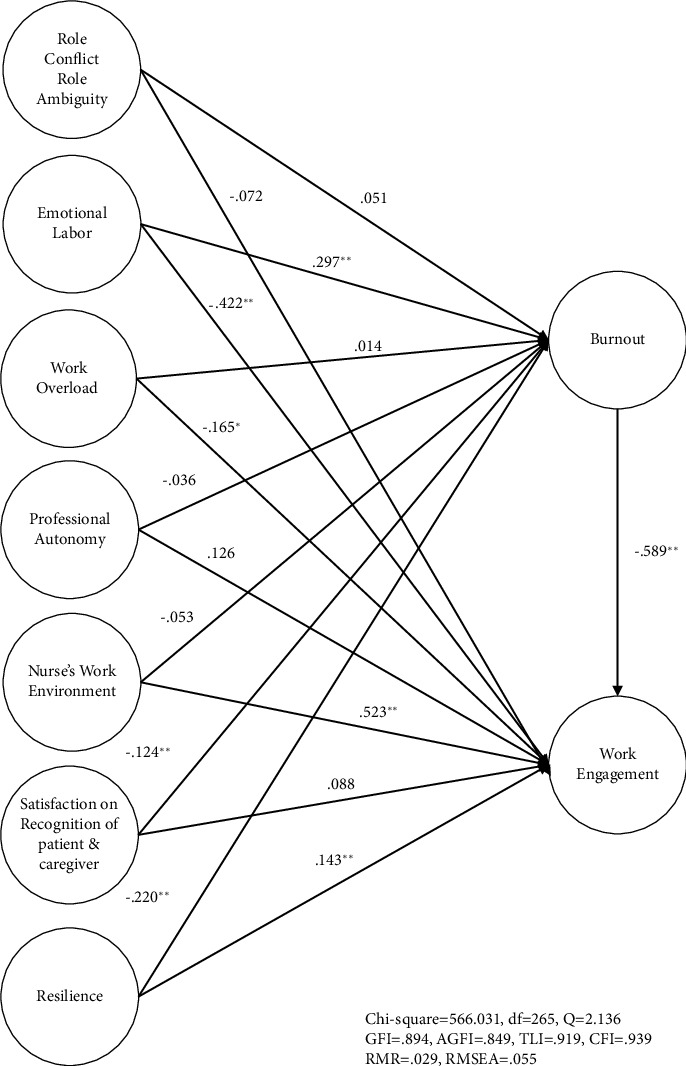
Structural equation modeling of the research model. ^∗^*p* < 0.05; ^∗∗^*p* < 0.01.

The proposed structural model proved to be a very good fit with *χ*^2^=566.031(*df*=265), *χ*^2^/*df*=2.136. The GFI was 0.894, AGFI was 0.849, TLI was 0.919, CFI was 0.939, RMR was 0.029, and RMSEA was 0.055, showing very high fit (Figure 2).

The variable with the greatest impact on nurses' burnout in Integrated Nursing-Care Service wards was emotional labor, followed by resilience and satisfaction with the recognition from patients and caregivers. Burnout was the most variable influencing nurses' work engagement in Integrated Nursing-Care Service wards, followed by nurses' work environment, emotional labor, work overload, and resilience (Table 2).

### 3.3. Results of Verification of Significance of Mediating Effect

Regarding the mediating effects, emotional labor, satisfaction with the recognition from patients and caregivers, and resilience had a significant effect on burnout. Regarding work engagement, emotional labor, work overload, and nurses' work environment had a significant effect on work engagement. Moreover, resilience and burnout had a significant effect on work engagement.

The indirect effects' results showed that burnout partially mediated the relationship between emotional labor and work engagement, fully mediated the relationship between satisfaction with the recognition from patients and caregivers and work engagement, and partially mediated the relationship between resilience and work engagement (Table 3).

## 4. Discussion

This study analyzed the factors affecting nurses' burnout and work engagement based on Xanthopoulou et al.'s [16] extended Job Demands-Job Resources model. The results of the structural model analysis confirmed that job demands, job resources, and personal resources affected burnout and work engagement. In addition, the proposed structural model, based on the upper and lower factors, showed a high degree of fit, confirming its high explanatory power. Based on these findings, we discuss the following points.

### 4.1. Factors Influencing Nurses' Burnout in Integrated Nursing-Care Service Wards

First, job demands had a positive effect on burnout. In particular, emotional labor had a significant effect on burnout, which is consistent with the findings of Kim et al. [39], Kim [40], and Hyun and Lee [41]. Emotional labor is common in interactive nursing work and can lead to increased burnout. Therefore, intervention programs and educational measures to reduce nurses' emotional labor are needed. Second, job resources of nurses in Integrated Nursing-Care Service wards had a negative effect on burnout. In particular, satisfaction with the recognition from patients and caregivers affected burnout. Patient and guardian recognition is an important variable nationwide related to the expansion of integrated nursing-care services. To improve this situation, publicity programs are needed to increase awareness and provide information about social nursing services. Third, resilience, a personal resource, had a negative effect on burnout, which is consistent with the findings of Kim [42], Yang and Gu [15], and Hyun and Lee [41]. As nurses' resilience is an acquired factor shaped by their personalities and stress management skills, a personal approach and organizational support are needed. It is necessary to develop various programs and provide stress management training to increase nurses' personal resilience and communication.

### 4.2. Factors Influencing Nurses' Work Engagement in Integrated Nursing-Care Service Wards

First, job demands of nurses in Integrated Nursing-Care Service wards had a negative effect on work engagement. In Choi's [43] study, a negative relationship between job demands and work engagement was reported, and among the subfactors, emotional labor and work overload had a negative effect on work engagement. The effects of emotional labor and workload on work engagement were reported in many domestic and international studies. As there is a lack of research on this topic, it is necessary to explore the relationship between job demands and work engagement in the special environment of nurses in Integrated Nursing-Care Service wards. Second, job resources had a positive effect on nurses' work engagement in Integrated Nursing-Care Service wards. The effect of nurses' work environment on work engagement was reported in previous studies; if nurses are fully aware of and satisfied with their job resources, they are more likely to experience positive work engagement. Therefore, efforts should be made to improve nurses' work environment and increase their awareness of job resources. Third, personal resources had a positive effect on work engagement. The greater the resilience, the higher the work engagement, which is consistent with the findings of Moon et al. [44] and Jang [45]. The effect of resilience on work engagement was confirmed in various studies, and recent studies related to the COVID-19 response showed that resilience plays an important role in the work experience of nurses. Therefore, programs aimed at improving resilience are needed to manage work engagement among nurses in Integrated Nursing-Care Service wards. Based on these findings, in order to effectively manage job burnout among nurses in Integrated Nursing-Care Service wards, efforts should be made to improve job demands, reduce emotional labor and work overload, and enhance job resources by improving nurses' work environment. In addition, programs aimed at enhancing nurses' personal resilience should be actively developed at the hospital organizational level.

### 4.3. Mediating Effects of Nurses' Burnout in Integrated Nursing-Care Service Wards

The structural model based on superordinate and subordinate factors confirmed the mediating effect of burnout on the relationship between job demands, job resources, and personal resources. Particularly, emotional labor and resilience had a partial mediating effect of burnout on subordinate factors, whereas satisfaction with the recognition from patients and guardians showed a full mediating effect. First, regarding the relationship between emotional labor and burnout, burnout showed a partial mediating effect, consistent with the findings of Shim [29], as nurses, especially in Integrated Nursing-Care Service wards, have more direct contact with patients, and the burden of emotional labor is greatly felt in the process of hiding their emotions and always treating them in a friendly manner to meet patients' and guardians' expectations of integrated nursing-care services. As a result, nurses are likely to experience burnout due to emotional labor, which affects their job enthusiasm. Therefore, emotional labor needs to be managed in Integrated Nursing-Care Service wards, and intervention programs are needed. Second, satisfaction with the recognition from patients and guardians showed a full mediating effect of burnout in its relationship with job commitment, which is different from the findings of Shim [29], where satisfaction with the recognition from patients and guardians did not directly affect job commitment but indirectly affected burnout. In this study, satisfaction with the recognition from patients and guardians in Integrated Nursing-Care Service wards mediated the relationship between burnout and work engagement, suggesting that good communication and interaction with patients and guardians, along with measures to reduce burnout, play an important role in mitigating work engagement. Third, resilience had an indirect effect on work engagement, and burnout mediated the effect of resilience on work engagement. Although the results of this study differ from those of previous studies, resilience seems to play a role in mitigating burnout and reducing work engagement depending on the characteristics of Integrated Nursing-Care Service wards.

Therefore, programs to enhance nurses' resilience and measures to manage burnout should be implemented. These findings suggest the need for intervention programs and organizational improvements that take into account the work characteristics of nurses in Integrated Nursing-Care Service wards, especially emotional labor, satisfaction with the recognition from patients and caregivers, and resilience. In doing so, we should focus on decreasing nurses' job burnout and increasing their overall job satisfaction.

### 4.4. Limitations

As this study was limited to nurses in tertiary hospitals, the results should be interpreted with caution, and it is recommended that the study be repeated considering the size of the hospital and various organizational characteristics.

## 5. Conclusions

This study used the JD-R model to examine the factors affecting burnout and work engagement among nurses in Integrated Nursing-Care Service wards. The structural model analysis substantiated the significant influence of job demands, job resources, and personal resources on both burnout and work engagement, affirming the robust fit of the proposed model and its applicability to nurses in specific healthcare settings. This study underscores the need for targeted interventions to address burnout management and enhance job enthusiasm among nurses. The key determinants of nurses' work engagement include burnout, nurses' work environment, emotional labor, work overload, and resilience. Notably, heightened emotional labor correlated with increased burnout. This study provides an effective framework for further research, particularly for the development of intervention programs aimed at improving nursing care quality and optimizing workforce management.

### 5.1. Implications for Nursing Management

This study represents a singular endeavor to substantiate a structural model that delineates the factors influencing the professional commitment of nurses working in Integrated Nursing-Care Service wards. The validated model serves as a pragmatic foundation for the formulation and assessment of nursing interventions in hospitals. Nursing management should prioritize the cultivation of a conducive work milieu, emphasizing the reinforcement of nurses' roles in hospitals. Furthermore, organizational support for initiatives targeting the amelioration of stress and burnout among nurses, coupled with enhancing individual resilience, is recommended for hospitals.

## Figures and Tables

**Table 1:** General characteristics of the participants (*N* = 375).

Characteristics	Category	*n*	%
Gender	Male	16	4.3
	Female	359	95.7

Age (years)	20–29	247	60.9
	30–39	87	23.2
	40–49	34	9.1
	≥50	7	1.9

Education	Associate bachelor	18	4.8
	Bachelor	322	85.9
	Master	30	8.0
	Others	5	1.3

Religion	Yes	137	36.5
	No	238	63.5

Marital status	Married	91	24.3
	Single	284	75.7

Position	Staff nurse	312	83.2
	Charge nurse	53	14.1
	Head nurse	10	2.7

Department	Medicine ward	203	54.1
	Surgical ward	172	45.9

Total clinical experience (years)	1–<5	194	58.6
	6–<10	63	19.0
	11–<15	29	8.8
	16–<20	21	6.3
	21≤	24	7.3

Experience in Integrated Nursing-Care Service wards (years)	1–<2	107	35.7
	3–<5	150	50.0
	6≤	43	14.3

**Table 2:** Summary of the test results for the hypotheses based on the research framework.

Path	Standardized coefficients	Unstandardized coefficients	Standard error	*t*	*p*	SMC (%)	Hypothesis supported
RC, RA ⟶ burnout	0.051	0.060	0.144	0.417	0.676	73.7	No
EL ⟶ burnout	0.297	0.354	0.120	2.950^*∗∗*^	0.003		Yes
WO ⟶ burnout	0.014	0.015	0.100	0.150	0.881		No
PA ⟶ burnout	−0.036	−0.040	0.057	−0.702	0.483		No
NWE ⟶ burnout	−0.053	−0.057	0.151	−0.377	0.706		No
SRPC ⟶ burnout	−0.124	−0.132	0.041	−3.220^*∗∗*^	0.001		Yes
Resilience ⟶ burnout	−0.220	−0.242	0.063	−3.841^*∗∗*^	0.000		Yes
RC, RA ⟶ WE	−0.072	−0.078	0.379	−0.206	0.837	85.7	No
EL ⟶ WE	−0.422	−0.454	0.159	−2.855^*∗∗*^	0.005		Yes
WO ⟶ WE	−0.165	−0.179	0.077	−2.325^*∗*^	0.021		Yes
PA ⟶ WE	0.126	0.135	0.149	0.906	0.366		No
NWE ⟶ WE	0.523	0.539	0.183	2.945^*∗∗*^	0.003		Yes
SRPC ⟶ WE	0.088	0.095	0.160	0.594	0.553		No
Resilience ⟶ WE	0.143	0.150	0.053	2.830^*∗∗*^	0.005		Yes
Burnout ⟶ WE	−0.589	−0.614	0.188	−3.266^*∗∗*^	0.001		Yes
*χ* ^2^ = 566.031 (*p*=0.000), *df* *=* 265, *Q* = 2,136							
GFI = 0.894, AGFI = 0.849, TLI = 0.919, CFI = 0.939, RMR = 0.029, RMSEA = 0.055 (90% CI = [0.049–0.061])							

RC, role conflict; RA, role ambiguity; EL, emotional labor; WO, work overload; PA, professional autonomy; NEW, nurses' work environment; SRPC, satisfaction with the recognition from patients and caregivers; WE, work engagement. ^*∗∗*^*p* < 0.01; ^*∗*^*p* < 0.05.

**Table 3:** Result of verifying significance of mediating effects on factors.

Path	Total effect	Direct effect	Indirect effect
RC, RA ⟶ burnout	0.051	0.051	
EL ⟶ burnout	0.297^*∗∗*^	0.297^*∗∗*^	
WO ⟶ burnout	0.014	0.014	
PA ⟶ burnout	−0.036	−0.036	
NWE ⟶ burnout	−0.053	−0.053	
SRPC ⟶ burnout	−0.124^*∗∗*^	−0.124^*∗∗*^	
Resilience ⟶ burnout	−0.220^*∗∗*^	−0.220^*∗∗*^	
RC, RA ⟶ WE	−0.102	−0.072	−0.030
EL ⟶ WE	−0.597^*∗∗*^	−0.422^*∗∗*^	−0.175^*∗*^
WO ⟶ WE	−0.173^*∗*^	−0.165^*∗*^	−0.008
PA ⟶ WE	0.147	0.126	0.021
NWE ⟶ WE	0.554^*∗∗*^	0.523^*∗∗*^	0.031
SRPC ⟶ WE	0.161^*∗*^	0.088	0.073^*∗*^
Resilience ⟶ WE	0.273^*∗∗*^	0.143^*∗∗*^	0.130^*∗*^
Burnout ⟶ WE	−0.589^*∗∗*^	−0.589^*∗∗*^	

RC, role conflict; RA, role ambiguity; EL, emotional labor; WO, work overload; PA, professional autonomy; NEW, nurses' work environment; SRPC, satisfaction with recognition from patients and caregivers; WE, work engagement. ^*∗∗*^*p* < 0.01; ^*∗*^*p* < 0.05.

## Data Availability

Data supporting the findings of this study are available upon request from the corresponding author. The data are not publicly available due to privacy or ethical restrictions.

## References

[1] Kwag W. H. (2015). The contents and problems of the pilot project for comprehensive nursing care service. *Medical policy forum*.

[2] Jung U. Y. (2019). *Patient Safety Culture and Safety Activities of Care-Helpers Working in Long Term Care Hospitals*.

[3] Oecd (2023). *OECDHealth Statistics 2023*.

[4] Statistics Korea (2016). *Korean Social Trends 2016*.

[5] Lee Y. M., Lee H. H., Jung J. H. (2018). A study about compassion fatigue, compassion satisfaction and burnout in comprehensive nursing care and general ward nurses. *Journal of Korean Clinical Nursing Research*.

[6] Kim Y. S., Park J. A., Seo E. K. (2019). A comparative study on the job stress, burnout and nursing performance of nurses in comprehensive nursing care service wards and nurses in general wards. *Stress: The International Journal on the Biology of Stress*.

[7] Kim J. H., Kim S. J., Park E. T., Jeong S. Y., Lee E. (2017). Policy issues and new direction for comprehensive nursing service in the national health insurance. *Journal of Korean Academy of Nursing Administration*.

[8] Kim M. S., Shin D. S., Choi Y. J. (2021). The influence of compassion fatigue, compassion satisfaction and nursing organizational culture on burnout in integrated nursing care units nurses. *The Journal of the Korea Contents Association*.

[9] Im J. A., Ko Y. (2021). Factors affecting turnover intention of nurses in comprehensive nursing care service wards: focusing on occupational stress, emotional labor, and burnout. *Korean Journal of Occupational Health Nursing*.

[10] Chowdhury S. r., Kabir H., Akter N. (2023). Impact of workplace bullying and burnout on job satisfaction among Bangladeshi nurses: a cross-sectional study. *Heliyon*.

[11] Nam H. A. (2014). Exploration of the relations with emotional labor of clinical nurses, social support, and job satisfaction. *Korean Society for Wellness*.

[12] Baek Y. M., Kim S. Y. (2020). Moderating and mediating effects of resilience in the relationship between work intensity, interpersonal conflict and burnout among nurses. *Journal of Korean Clinical Nursing Research*.

[13] Liu F., Zhang Z., Liu S., Zhang N. (2021). Examining the effects of brief mindfulness training on athletes’ flow: the mediating role of resilience. *Evidence-based Complementary and Alternative Medicine*.

[14] Oh A. R., Lee T. H. (2016). A Study on employees’ positive psychological capital and job engagement and job behavior. *Korean Management Consulting Review*.

[15] Yang E. O., Gu M. O. (2022). A structural model for burnout and work engagement of nurses in long-term care hospitals: application of the expanded job demand-job resources model. *Journal of Korean Gerontological Nursing*.

[16] Xanthopoulou D., Bakker A. B., Demerouti E., Schaufeli W. B. (2007). The role of personal resources in the Job Demands-Resources model. *International Journal of Stress Management*.

[17] Keyko K., Cummings G. G., Yonge O., Wong C. A. (2016). Work engagement in professional nursing practice: a systematic review. *International Journal of Nursing Studies*.

[18] Woo J. (2012). *Structural Equation Model Concepts and Understanding*.

[19] Rizzo J. R., House R. J., Lirtzman S. I. (1970). Role conflict and ambiguity in complex organizations. *Administrative Science Quarterly*.

[20] Kim H. W. (2014). A study on the effect of convalescent hospital nurses’ role ambiguity and role conflict: the Mediating job stress and regulation effect of justice.

[21] Morris J. A., Feldman D. C. (1996). The dimensions, antecedents, and consequences of emotional labor. *Academy of Management Review*.

[22] Kim M. J. (1998). Effects of the hotel employee’s emotional labor upon the job - related attitudes. *Journal of Tourism Sciences*.

[23] van Veldhoven M. J. P. M., Prins J., van der Laken P. A., Dijkstra L. (2015). *QEEW2.0: 42 Short Scales for Survey Research on Work, Well-Being and Performance*.

[24] Lim Y. Y. (2018). *Nurses’ Intention to Leave and Organizational Citizenship Behavior: Verification of the Job Demands-Job Resources Model*.

[25] Schutzenhofer K. K. (1983). The development of autonomy in adult women. *Journal of Psychosocial Nursing and Mental Health Services*.

[26] Han C. B., Moon H. J. (1996). A study on role conception and job satisfaction of clinical nurses. *Korean Journal of Nursing Administration*.

[27] Kim I. W. (2005). *A Structural Model on Head Nurses’ Leadership*.

[28] Kim J. K., Kim M. J., Kim S. Y., Yu M., Lee K. A. (2014). Effects of general hospital nurse’s work environment on job embeddedness and burnout. *Journal of Korean Academy of Nursing Administration*.

[29] Shim S. S. (2015). *Structural Equation Modeling on Burnout and Organizational Commitment in Hospital Nurses*.

[30] Stamps P. L., Piedmont E. B., Slavitt D. B., Haase A. M. (1978). Measurement of work satisfaction among health professionals. *Medical Care*.

[31] Park H. T. (1996). Transformational and transactional leadership styles of the nurse administrators and job satisfaction, organizational commitment in nursing service.

[32] Choi A. Y. (2012). A study on determinant of women volunteer in consumer groups: focused on the satisfactions, interpersonal skills and recognize and compensation factors.

[33] Connor K. M., Davidson J. R. (2003). Development of a new resilience scale: the Connor‐Davidson resilience scale (CD‐RISC). *Depression and Anxiety*.

[34] Baek H. S. (2010). *Reliability and Validity of the Korean Version of the Connor-Davidson Self-Resilience Scale Validity Study of the Korean Version of the Connor-Davidson Self-Resilience Scale*.

[35] Demerouti E., Nachreiner F. (1998). Aufsätze-Zur Spezifität von burnout für dienstleistungsberufe: fakt oder artefakt. *Zeitschrift fur Arbeitswissenschaft*.

[36] Choi K. J. (2007). Factors influencing on burnout of the nurses in hospitals.

[37] Schaufeli W. B., Bakker A. B., Salanova M. (2006). The measurement of work engagement with a short questionnaire: a cross-national study. *Educational and Psychological Measurement*.

[38] Baek S. J. (2016). *The Structural Relationship Among Job Crafting Behavior, Work Engagement and Change-Oriented Behavior*.

[39] Kim J. S., Jeong S. Y., Kim S. H., Kim J. O. (2014). Predictors of emotional labor and job stress on burnout of nurses in long-term care hospitals. *Journal of Korean Gerontological Nursing*.

[40] Kim I. S. (2015). The role of emotional labor strategies based on job demand- resource theory. *The Journal of the Korea Contents Association*.

[41] Hyun I. S., Lee S. Y. (2020). Mediating effects of Burnout between emotional labor and resilience for nurses in long-term care hospitals. *Journal of the Korea Academia Industrial cooperation Society*.

[42] Kim W. S. (2017). *A Structural Model of Burnout Among Nursing Home Nurses Performing End-Of-Life Care*.

[43] Choi S. (2012). *The Influence of Working Environment of Schools on Burnout and Engagement Among Teachers*.

[44] Moon I. O., Park S. K., Jung J. M. (2013). Effects of resilience on work engagement and burnout of clinical nurses. *Journal of Korean Academy of Nursing Administration*.

[45] Chang S. I. (2019). *Korean Review of Corporation Management*.

